# Triggers for Palliative Care Referral in Pediatric Oncology

**DOI:** 10.3390/cancers13061419

**Published:** 2021-03-19

**Authors:** Andrea Cuviello, Catherine Yip, Haven Battles, Lori Wiener, Renee Boss

**Affiliations:** 1 Department of Oncology, St Jude Children’s Research Hospital, Memphis, TN 38105, USA; 2 Department of Medicine, Johns Hopkins School of Medicine, Baltimore, MD 21218, USA; cyip5@jhmi.edu (C.Y.); rboss1@jhu.edu (R.B.); 3 Pediatric Oncology Branch, National Cancer Institute, National Institutes of Health, Bethesda, MD 21218, USA; haven.battles@nih.gov (H.B.); wienerl@mail.nih.gov (L.W.); 4 Department of Medicine, Johns Hopkins Berman Institute of Bioethics, Baltimore, MD 21218, USA

**Keywords:** palliative care, pediatric oncology, quality of life, symptom management, screening tool, trigger

## Abstract

**Simple Summary:**

Palliative care (PC) can improve the quality of life for pediatric cancer patients, yet these services remain underutilized, with referrals occurring late in the disease course or not at all. We previously described the patient and family characteristics that diverse pediatric oncology providers agree should be high yield triggers for PC referral in pediatric cancer patients. The current study examined how often those triggers were associated with a completed PC consult for a cohort of 931 patients. We discovered that PC referrals occur very infrequently and patients with stated triggers often do not get referred. These findings help support the need for a screening tool to standardize PC integration and improve care.

**Abstract:**

Palliative care (PC) integration into the care of pediatric oncology patients is growing in acceptance and has been shown to improve the quality of life of children with cancer. Yet timing for referrals and referral practices remain inconsistent, and PC remains underutilized. We conducted a retrospective chart review of pediatric oncology patients treated at an academic institution between January 2015 to November 2018. Data collected included demographics, disease and therapy characteristics, and consultation notes, specifically documenting existence of predetermined “high yield triggers” for PC consultation. Among 931 eligible patients the prevalence of PC consultation was 5.6% while approximately 94% of patients had at least 1 trigger for PC consultation. The triggers that more often resulted in PC consultation included: symptom management needs (98%; *n* = 51) high-risk disease (86%; *n* = 45), poor prognosis (83%; *n* = 43), multiple lines of therapy (79%; *n* = 41) and a documented ICU admission (67%; *n* = 35). Our findings suggest that the high yield triggers for palliative care consultation that pediatric oncologists identify as important are not translating into practice; incorporating these triggers into a screening tool may be the next step to improve early PC integration.

## 1. Introduction

Palliative care (PC) has been shown to improve quality of life, symptom distress, communication needs and end of life care for pediatric patients and has consequently become increasingly accepted in the realm of pediatric oncology [1,2,3,4]. However, actual consultation continues to occur late in the disease course [5,6] even as advances in pediatric oncology have shifted the paradigm from cure-based to chronic illness, underscoring the relevance of earlier PC involvement [7]. Consultative pediatric PC is increasingly available at centers offering pediatric secondary and tertiary level care [8]. Issues like difficult-to-manage symptoms, complex family dynamics, and challenging care decisions regarding life-sustaining treatment and potential hospice involvement regularly prompt PC referrals across multiple pediatric specialties.

Many pediatric oncologists feel that PC delivery is a part of their role with patients and their families [9]. Despite this, time constraints, limited formalized PC training and education, nuances of the therapeutic relationship, and complex physical and psychological symptoms may warrant referral for PC subspecialty care [10,11,12]. Wentlandt et al. [13]. reported that the personal characteristics, beliefs and attitudes of the primary provider will inevitably reflect the timing of PC consultation. Yet referrals that are made late in the disease course may cause patients and families to suffer unnecessarily. And given the limitation of PC resources in many hospitals across the country [14], it is important for the early identification of patients to allow for time for PC subspecialists to develop relationships and meaningfully contribute to goals of care. In a recent semi-structured interview study pediatric oncology provider identified patient triggers for early PC referral and endorsed the development of a standardized screening tool with these triggers to facilitate earlier PC involvement [15]. This study aimed to determine how often patients at a major cancer center had the documented triggers for PC consultation, and among them, how many then received a PC consult.

## 2. Materials and Methods

We conducted an IRB exempt, retrospective chart review of pediatric oncology patients aged 0–26 years treated at an academic institution between January 2015 to November 2018. Reasons for exclusion included (1) patients who were candidates for bone marrow donation and thus did not have an oncologic diagnosis themselves, (2) patients who did not have an oncological diagnosis, (3) patients who had an oncological diagnosis but did not receive treatment at the study institution and therefore were never clinically followed at the institution after initial evaluation, or (4) patients who were a part of the survivorship program, as these patients would have been more than 5 years off of therapy and may have preceded the era of PC services at the study institution. Data collection was conducted by two study members (AC and CY) utilizing independent medical record abstraction, collaborative review, and consensus building. Demographic variables collected included age, gender, primary oncology attending physician and fellow, diagnosis, and date of death (where applicable). Trigger related data collection was divided into three categories: (1) Disease-related triggers including prognosis, high risk disease, and comorbidities at the time of diagnosis (2) therapy-related triggers including number of lines of therapy, number of relapses, enrollment into a phase 1 study, history of BMT, and ICU admission; and (3) symptom-related triggers including subspecialty consultation specifically, pain team, palliative care and psychology or psychiatry involvement.

For the ease of analysis, diagnoses were grouped into broader categories labeled as group 1–7, representing hematological malignancies, sarcomas, CNS tumors, histiocytic disorders, red cell disorders, immunodeficiencies, and neuroendocrine tumors respectively. Of note, red cell disorders and immunodeficiencies often receive bone marrow transplantation as curative therapy, a previously identified PC consultation trigger, and thus were included in this study. Parameters for defining high-risk disease, poor prognosis, intense treatment and symptom management were based on literature review. High-risk disease was defined as any disease that was metastatic at the time of diagnosis or assigned to a high-risk treatment protocol or was refractory in nature. Prognosis calculations were based on patient history, age, laboratory evaluations, disease staging, initial disease response where applicable, and defined as an event-free survival <70%. Intense treatment included patients who underwent BMT, received inpatient chemotherapy at minimum every two weeks, participated in an experimental phase 1 trial, or received three of the following: chemotherapy, immunotherapy, surgery, or radiation. Symptom management issues were identified as the presence of pain, nutritional issues and mental health needs that were secondary to treatment. A trigger was deemed present if the symptom management need required a subspecialty consult at any given time, was mentioned in the most recent physician progress note, or were active on the patient’s problem list. All triggers were only counted once.

Descriptive analyses are provided. For all outcomes comparing those who did and did not receive a palliative care consult, Pearson chi-square analyses were utilized. No cell sizes were smaller than five, so Fisher’s Exact Test was not utilized. All analyses were conducted using SPSS version 21.

## 3. Results

### 3.1. Patient Demographics

A total of 931 patients met study eligibility. Reflecting the chronic burden of pediatric cancer, 819 patients (88%) were alive at time of chart review and 559 patients (60%) had comorbidities at time of cancer diagnosis (Table 1). As anticipated with cancer incidence based on disease type, over a third of patients (*n* = 354, 38%) had hematological malignancies, 158 patients (17%) were diagnosed with sarcomas, and 149 patients (16%) with a CNS tumor.

### 3.2. PC Consultation Findings

The overall prevalence of PC consultation was 5.6%. There were no statistically significant differences noted between gender and the presence of pre-oncological comorbidities. Patients who died were more likely to have had PC consultation (13.6% vs. less than 7% for other diagnostic categories, χ^2^ = 28.7, *p* < 0.001). 

Of the disease-related triggers, patients with high-risk disease (8.6% vs. 1.7%, χ^2^ = 20.5, *p* < 0.001), poor prognosis (15.7% vs 1.4%, χ^2^ = 75.2, *p* < 0.001), or a sarcoma (13.6% vs. less than 7% for other diagnostic categories, χ^2^ = 28.7, *p* < 0.001) were more likely to receive PC consultation. Children with hematological malignancies, CNS tumors, histiocytic disorders, red cell disorders or immunodeficiencies or the presence of comorbidities was not associated with PC consultation (Table 1).

**Figure 1:**
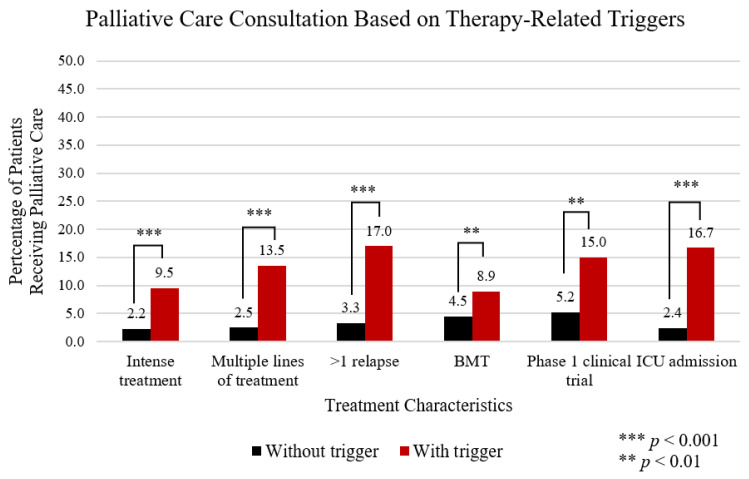
Demonstrates a comparison of all patients with a PC consultation in relation to the presence (red bar) or absence (black bar) of treatment-related PC triggers.

In regard to therapy-related triggers, 46.5% received intense treatment, 27.8% of patients had multiple lines of therapy, 16.4% experienced more than one relapse, 25.3% had at least one bone marrow transplant, 4.3% participated in a phase 1 clinical trial and 22.4% of patients were admitted to the ICU. All were associated with higher rates of PC consultation (Figure 1).

**Figure 2:**
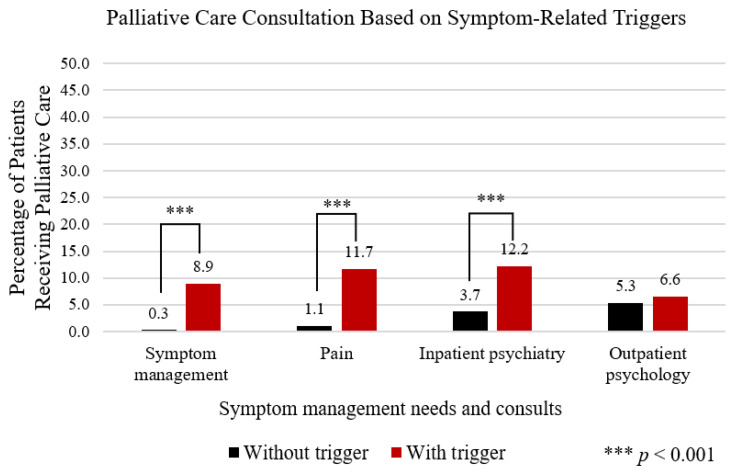
Demonstrates a comparison of all patients with a PC consultation in relation to the presence (red bar) or absence (black bar) of symptom-related PC triggers.

Among the sample study, 61.7% of patients met the definition of symptom management needs. Of these patients, 42.3% were referred to the pain team, 22% and 22.8% were referred to inpatient psychiatry and outpatient psychology (Figure 2). Patients were statistically more likely to have PC consultation if they were also referred to the pain team or inpatient psychiatry (Figure 2).

### 3.3. Overall Trigger Associated PC Consultation Patterns

Of the 5.6% (*n* = 52) of patients who received PC consultation, 98% (*n* = 51) had a documented trigger of symptom management needs, 86% (*n* = 45) had high-risk disease, 83% (*n* = 43) had documentation of poor prognosis, 79% (*n* = 41) received mutliple lines of therapy, 67% (*n* = 35) had an ICU admission documented throughout their treatment course, 65% (*n* = 34) were deceased at the time of review, 50% (*n* = 26) had relapsed disease, 40% (*n* = 21) required a BMT and 11% (*n* = 6) enrolled in a phase 1 clinical trial.

Interestingly, all patients who received PC consults had 4 or more triggers documented. Among the entire study population 53 patients or 5.7% had no documented triggers, 116 patients (12%) had only 1 documented trigger, 473 patients (51%) had between 2 and 4 triggers, and 289 patients (31%) had 5 or more triggers. The prevalence of PC consultation among those with 5+ documented triggers was 16%.

## 4. Discussion

Oncologists have identified specific reasons to consult PC including disease, therapy and symptom-related triggers. We looked at 931 patients, 94% of whom had at least 1 pre-identified trigger, and yet only 52 patients (5.6%) were referred to PC. The triggers most frequently associated with PC consultation were disease type, poor prognosis, high risk or relapsed disease, intense therapy, BMT, phase 1 enrollment, and significant symptoms which seem to prompt PC consultation most consistently, albeit inconsistently. With an overall PC consultation prevalence of 5.6%, our study suggests that PC remains extremely underutilized despite its noted potential to improve the physical, emotional and spiritual well-being of a child undergoing treatment for a life-threatening illness [2,10,16,17,18,19,20,21]. Additionally, it was not until patients reached four or more documented triggers that PC was consulted. Demographics such as age or gender did not play a role in PC referral practices in our findings, although have been previously cited as triggers for PC consultation [21]. Of note, the age range in this patient population reflects the institution’s practice of caring for all pediatric cancers in patients aged 0–26 years. Interestingly, patients with CNS tumors, which generally carry a poor prognosis, or who were found to be deceased at the time of review had extremely low rates of PC consultation, for which further exploration with future studies may be helpful. Together, our findings suggest that a starting point for increasing PC involvement is a trigger-based tool to help pediatric oncology providers improve the integration of PC into the care of children with cancer.

Previous literature highlights the predominant opinion that PC is consulted “too late” in the realm of pediatric oncology [6,15,22,23] and our group has previously shown that 84% of interdisciplinary pediatric oncology providers thought that a screening tool could help overcome this problem and increase early PC involvement. Providers in that study identified specific “high yield” trigger criteria for a screening tool, including poor prognosis, symptom management needs, comorbidities and psychosocial needs [15]. Additional literature-based triggers for early PC integration include the need for BMT, no therapeutic options remaining and disease progression [16,17,24]. Combining these triggers in the current study, some of these disease, therapy, and symptom characteristics were associated with a statistically significant increased likelihood for PC consultation, suggesting that a screening tool could be helpful. However, it was not until patients had several (4+) documented triggers that PC involvement was initiated.

It is well known that referral patterns to PC can be influenced by a number of factors including a provider’s individual practice, based on individual definitions and knowledge of PC, emotional influences, such as long-term and invested relationships with patients and their families, and socially acceptable influences [21,25,26]. A survey of pediatric oncologists found that 74% reported reluctance in consulting PC as this felt like they were “giving up” on their patients [27]. Additionally, as medicine continues to make advances in the treatment of childhood cancer, prognostication becomes more uncertain, and may contribute to late PC referrals [23,28,29,30,31,32]. In one of our earlier studies, we found that providers were able to theorize ideal triggers for PC referral better than they were able to identify them in a clinical case scenario [15]. For example, comorbidities in our study were not found to increase the likelihood of PC consultation yet were previously cited by oncologists as a potential trigger for PC [15]. This is particularly important as the disease paradigm for cancer treatment shifts from a cure-based model to a chronic illness model thereby increasing the likelihood for the presence of comorbidities in this patient population as a result of disease or therapy related complications. Overall, our findings in this paper reinforce this gap between theoretical and practical referral practices, emphasizing the need for the creation of an optimal referral model or tool.

In the adult oncology realm, the National Comprehensive Cancer Network (NCCN) recommends that all patients with cancer be screened for PC needs at their initial visit and then subsequently as clinical needs arise [33]. The guideline for screening encompasses 6 domains: pain and other symptoms, psychological distress, comorbid physical and psychosocial conditions, treatment options, patient or family concerns and decision making and finally, prognosis [33,34]. Notably, several of these domains are highlighted as triggers for PC consultation by pediatric oncologists as well. One study by Glare and Chow demonstrated that this screening tool was both feasible and valid at identifying adult oncology patients in need of subspecialty PC services [34]. This has yet to be studied in a similar manner in pediatric oncology.

Commonly, reported barriers to PC involvement revolve around lack of communication and resources, increased need for education, and systems-based issues [5,10,15,30]. Our study offers the foundation for a potential solution to assist with systems-based barriers, specifically, the standardization of referral practices to PC through a screening tool. This could be implanted within an electronic health system to not only remove individual practitioner bias (i.e., key words such as relapsed disease or BMT could trigger the need for completion of a PC screening tool), but also to improve workflow and enhance interdisciplinary team communication. Ideally, this would add an additional benefit of potentially alleviating some of the distress oncologists experience when discussing the involvement of PC with patients and families through a mode of standardization and naturalization [35].

While the use of a screening tool to guide PC referrals is not yet widely adopted, evidence supports early PC integration as the gold standard for pediatric oncology patients [17,36,37,38] and is supported by organizations such as the American Society of Clinical Oncology (ASCO), the American Academy of Pediatrics, and the World Health Organization (WHO) [39,40,41,42]. Currently, the field of pediatric PC lacks a standardized framework for early PC referral and would require the expertise of influential stakeholders as well as internal and external validity for the development of a such a screening tool [43]. We hope that the unique findings in this paper demonstrate that disease, therapy and symptom related characteristics of a patient’s journey with cancer can help guide providers with early PC integration.

Several strengths and limitations exist for this study. This was a retrospective study completed at a single institution, which may not allow for generalizability. Data collection was completed in 2018 with the completion of the primary study member’s fellowship training and may not reflect current PC consultation practices. However, the cohort of eligible charts reviewed is respectable and the triggers for PC consultation used to determine data abstraction information were provided by pediatric oncology physicians at the same institution. Lastly, our study did not examine the relationship between timing of PC consultation and each respective trigger. Further exploration into referral practices would be helpful to better inform the development of a standardized PC screening tool in pediatric oncology.

Overall, our study findings are exceedingly insightful as they capture the referral patterns of pediatric oncologists in a practical sense. In addition, 94% of patients reviewed in this study possessed at least one pre-identified trigger for PC consultation, yet the overall prevalence for PC consultation was <5%, suggesting that there remains a gap between our theoretical and actual referral practices for PC.

## 5. Conclusions

This study uses previously cited high yield triggers for PC consultation reported by pediatric oncologists to compare actual PC referral patterns. We demonstrate that theoretic triggers for PC referral are not consistently being implemented in practice and perhaps contributing to late PC involvement. Future investigations directed at further development and validity testing for a PC screening tool would be helpful to minimize variability in PC referral patterns and to standardize practice.

## Figures and Tables

**Table 1:** Characteristics of pediatric oncology patients who did and did not receive Palliative Care consultation between January 2015 and November 2018.

General Demographics	Patients without PC Consult (%) *n* = 879	Patients with PC Consult (%) *n* = 52
Sex		
Male	518 (58.9)	36 (69.2)
Female	361 (41.1)	16 (30.8)
Age		
<18 year	566 (64.4)	29 (55.8)
≥18 year	313 (35.6)	23 (44.2)
Diagnosis Category		
Hematological malignancies	343 (39.0)	15 (28.8)
Sarcomas	140 (15.9)	22 (42.3)
CNS tumors	150 (17.1)	2 (3.8)
Histiocytic disorders	32 (3.6)	0 (0)
Red cell disorders	59 (6.7)	3 (5.8)
Immunodeficiencies	28 (3.2)	1 (1.9)
Neuroendocrine tumors	127 (14.4)	9 (17.3)
Vital Status		
Alive	805 (91.6)	18 (34.6)
Deceased	74 (8.4)	34 (65.4)
High Risk Diagnosis		
Yes	479 (54.5)	45 (86.5)
No	400 (45.5)	7 (13.5)
Poor prognosis		
Yes	231 (26.3)	43 (82.7)
No	648 (73.7)	9 (17.3)
Comorbidities		
Yes	525 (59.7)	30 (57.7)
No	354 (40.3)	22 (42.3)

Abbreviations: CNS, central nervous system; PC, Palliative Care.

## Data Availability

The data presented in this study are available on request from the corresponding author. The data are not publicly available due to HIPPA protection laws.
